# Family Associations in a Breeding Colony of Critically Endangered Hooded Vulture (*Necrosyrtes monachus*) in the Lowveld of South Africa

**DOI:** 10.1002/ece3.70606

**Published:** 2024-11-21

**Authors:** Rynhardt Le Roux, Lindy J. Thompson, Bettine Jansen van Vuuren, Sandi Willows‐Munro

**Affiliations:** ^1^ Centre for Functional Biodiversity, School of Life Science University of Kwazulu‐Natal Pietermaritzburg South Africa; ^2^ Southern African Wildlife College Hoedspruit South Africa; ^3^ Centre for Ecological and Genomics and Wildlife Conservation, Department of Zoology University of Johannesburg Auckland Park South Africa

**Keywords:** hooded vulture, *Necrosyrtes monachus*, nesting, Olifants River, South Africa

## Abstract

Numbers of Critically Endangered Hooded Vultures (*Necrosyrtes monachus* Temminck 1823) are declining across their distribution. The range‐edge population in South Africa is one of the smallest populations with only 100–200 mature individuals. In South Africa, Hooded Vultures nest solitarily in loose colonies (mean distance between nests 0.76 km) along water courses. Basic ecological information, such as breeding behaviour, is still lacking for the species. In this study, we examine the relatedness and nest turnover of nesting individuals along the Olifants River and other locations in the Lowveld of South Africa by sampling nests over five consecutive years. A key hypothesis tested is whether communal roosting sites function as information‐sharing hubs, a phenomenon that has been seen in Cape Vultures (*Gyps coprotheres*) and other cliff‐nesting vultures. Theory suggests that information sharing occurs more frequently between closely related individuals. If true, we expect distance between nests and genetic relatedness to be positively correlated and individuals to use the same nests over multiple years. Naturally moulted feathers (*n* = 108) were collected below nests over five consecutive years, and 14 microsatellite loci markers were used to measure genetic relatedness. Mantel tests performed correlating distance between nests to relatedness coefficient estimators TrioML (*r* = 0.032, *R* ^2^ = 0.001, *p* = 0.224) and LynchRD (*r* = 0.007, *R* ^2^ = 0.00005, *p* = 0.403), found no statistical correlation. The Mantel test performed using Nei's genetic distance and distance between nests did show a negative correlation (*r* = −0.108, *R* ^2^ = 0.0117, *p*‐value = 0.012), indicating that individuals that were more closely related tended to breed further away. No nest reuse was found in this study. We thus believe that these loose colonies do not act as food‐finding hubs, but rather that the Olifants River is an important breeding site for these birds.

## Graphical abstract

Numbers of critically endangered Hooded Vultures (*Necrosyrtes monachus* Temminck 1823) are declining across their distribution. Basic ecological information, such as breeding behaviour, is still lacking for the species. In this study we examine the relatedness and nest turnover of nesting individuals along the Olifants River and other locations in the Lowveld of South Africa by sampling nests over five consecutive years. No correlations was found between geographic distance between nests and relatedness coefficient estimators. No nest reuse was found in this study. We thus believe that these loose colonies do not act as food finding hubs, but rather that the Olifants River is an important breeding site for these birds.

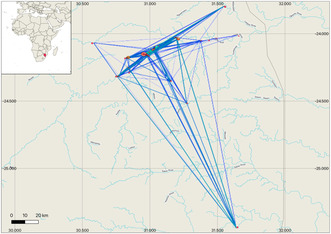

## 1 Introduction

Communal nesting is a common phenomenon observed in many bird species (Ward and Zahavi 1973; Hagan and Walters 1990). Colonial living may confer a fitness advantage to individuals by lowering thermoregulation demands, lowering the risk of predation and increasing foraging success. In contrast, communal nesting may arise as a consequence of resource constraints either through limited availability of suitable nesting sites or proximity to food (Ward and Zahavi 1973; Van Overveld et al. 2020; Penttinen et al. 2024) and may occur even when there are no obvious fitness benefits to individuals (Ward and Zahavi 1973).

The information center hypothesis suggests that colonial living is linked to increased foraging success as information on the location of patchily distributed food is shared between members of the colony (Ward and Zahavi 1973; Hagan and Walters 1990). This may be particularly advantageous for species that rely on abundant but transient food sources. As such communal roosts or breeding colonies of scavengers such as vultures may act as these ‘food finding information hubs’ allowing for the exchange of information regarding potential food sources (Ward and Zahavi 1973; Dermody, Tanner, and Jackson 2011). This may, at least in part, explain the high levels of relatedness often found within colonies (Erwin 1978; Waltz 1982), with close relatives more likely to tolerate the cost of sharing food by increasing their inclusive fitness (Hamilton 1964). Research on other African vulture species, such as the Cape Vulture (*Gyps coprotheres*), suggests that communal roosts or colonies do act as important food‐finding centres for these obligate scavengers, where individuals communicate sources of potential food and transfer other important information (Dermody, Tanner, and Jackson 2011; Martens et al. 2020).

Unfortunately, limited information is available on many aspects of life history for most species of African vultures, including the factors that affect reproduction in the wild, and the reasoning behind loose colony formation when breeding in some species such as the Hooded Vulture, *Necrosyrtes monachus* (Monadjem et al. 2016).

This study thus aims to shed some light on the life history of Hooded Vulture colonies in South Africa, by assessing if Hooded Vulture colonies in South Africa act as food‐finding information hubs. Therefore, if Hooded Vulture colonies are operating as ‘food finding information hubs’, we hypothesise that individuals nesting close to each other should be more closely related and individuals should use the same nests over multiple years to increase overall fitness. These predictions were tested in this study by determining the relatedness of individuals nestling along a section of the Olifants River in the Limpopo Province, South Africa, using genetic data from fourteen microsatellites. Samples collected from the same nest over many years were used to determine if the same individuals were using the same nest over multiple years.

## 2 Materials and Methods

### 2.1 Study Species

The Hooded Vulture (*Necrosyrtes monachus* Temminck 1823) is widespread across sub‐Saharan Africa (Ferguson‐Lees and Christie 2001; eBird 2021). Although the species is widely distributed, at a local scale individual birds are resident and generally sedentary, with some limited dispersal by non‐breeders and immature birds (Ferguson‐Lees and Christie 2001; Reading et al. 2019; Thompson et al. 2020). The species has experienced population declines across most of its distribution as a result of anthropogenic factors, such as poisoning (intentional and unintentional), electrocutions and/or collisions with pylons and powerlines, and poaching for use in traditional medicine (Ogada and Buij 2011; Ogada, Keesing, and Virani 2012; Odino, Imboma, and Ogada 2014; Saidu and Buij 2018; Williams, Ottosson, and Deikumah 2021). As a result of dramatic contemporary population declines, this species has been flagged by the IUCN and is listed as critically endangered (BirdLife International 2022). Thus, an improved understanding of the population and breeding dynamics of the South African Hooded Vulture population could benefit conservation efforts (Reading et al. 2005; Carrete et al. 2006; Margalida et al. 2008; Zuberogoitia et al. 2008). For example, an increased understanding of nest fidelity in this species could help guide whether nesting trees/habitats need to be protected (Thompson and Blackmore 2020).

Although large flocks of Hooded Vultures are common in West Africa (Gbogbo and Awotwe‐Pratt 2008; Jallow et al. 2016, 2022; McLachlan and Liversidge 2016), the range‐edge South African population is much smaller, including only ±100–200 mature individuals (Taylor, Peacock, and Wanless 2015). The conservation value of these small peripheral populations is debatable, as smaller populations tend to lose genetic diversity quickly through the processes of genetic drift and inbreeding. On the other hand, these range‐edge populations may act as important reservoirs of rare alleles and may provide important information on how the species may react under different climate change scenarios (Sagarin and Gaines 2002; Eckert, Samis, and Lougheed 2008; Nowell, Wang, and Smith 2022). In the 1980s, the breeding population of South Africa was estimated to be lower than 50 pairs, but since the early 2000s, the breeding population has steadily increased (Roche 2006). This increase could reflect a more intense research focus rather than an increase in population size (Daneel 1984; Roche 2006; Monadjem et al. 2016). Understanding the dynamics of this South African population of Hooded Vultures could provide important information for the conservation of the species. In particular, important information on breeding ecology is needed to improve conservation plans.

Hooded Vultures are monogamous and may be seen around the same nest all year round but produce chicks only once per year. If the breeding attempt fails early in the breeding season, a second breeding attempt may occur. Unlike other tree‐nesting vulture species that generally nest on the tops of trees, Hooded Vultures usually nest within the canopies of well‐foliaged trees. In South Africa, the tree species that are often utilised are jackal‐berry trees (*Diospyros mespiliformis*) and common cluster figs (*Ficus sycomorus*) along water courses (Mundy et al. 1992; Roche 2006; Monadjem et al. 2016; Fern, Thompson, and Downs 2022). That their nesting trees are usually very densely foliated makes direct observation of Hooded Vultures' nesting behaviour difficult. Although solitary nesters in South Africa, Hooded Vultures often nest in close proximity forming loose colonies (Monadjem et al. 2016). A study of twelve Hooded Vulture nests along the Olifants River Privat Nature Reserve (PNR) (24°09′46″ S, 31°01′27″ E) in Limpopo Province, South Africa, found that the mean distance between active nests was 0.76 km, with some active nests being just 60 m apart (Monadjem et al. 2016). Nest‐site selection could be adaptive in the species as nests often remain active for many years. For example, one of the first breeding records available for the Kruger National Park (South Africa) was a nest in Bangu Gorge recorded in 1967, and this same nest was still active 20 years later (Daneel 1984; Roche 2006; Monadjem et al. 2016). It is unclear whether a pair of vultures use the same nest throughout their life or whether offspring will take over their parents' nest.

### 2.2 Sampling

Ethical clearance for this project was granted by the Animal Research Ethics Committee of the University of KwaZulu‐Natal (AREC/094/015PD, AREC/022/020). A Section 20 permit (ref 12/11/1/5; 2104NC) and a veterinary permit (ref MJV GR 76/22) were obtained for the collection and movement of samples. Fieldwork was done with provincial research permits for Limpopo Province (ZA/LP/HO/2937—Oct 2015—Oct 2016, ZA/LP/80214—Jan 2017 to Jan 2018, ZA/LP/90993—Aug 2018 to Aug 2019, ZA/LP/100606—Oct 2019 to Oct 2020) and for Mpumalanga Province (permit no. MPB. 5557—2016, 5581—2017, 5619—2018).

**FIGURE 1:**
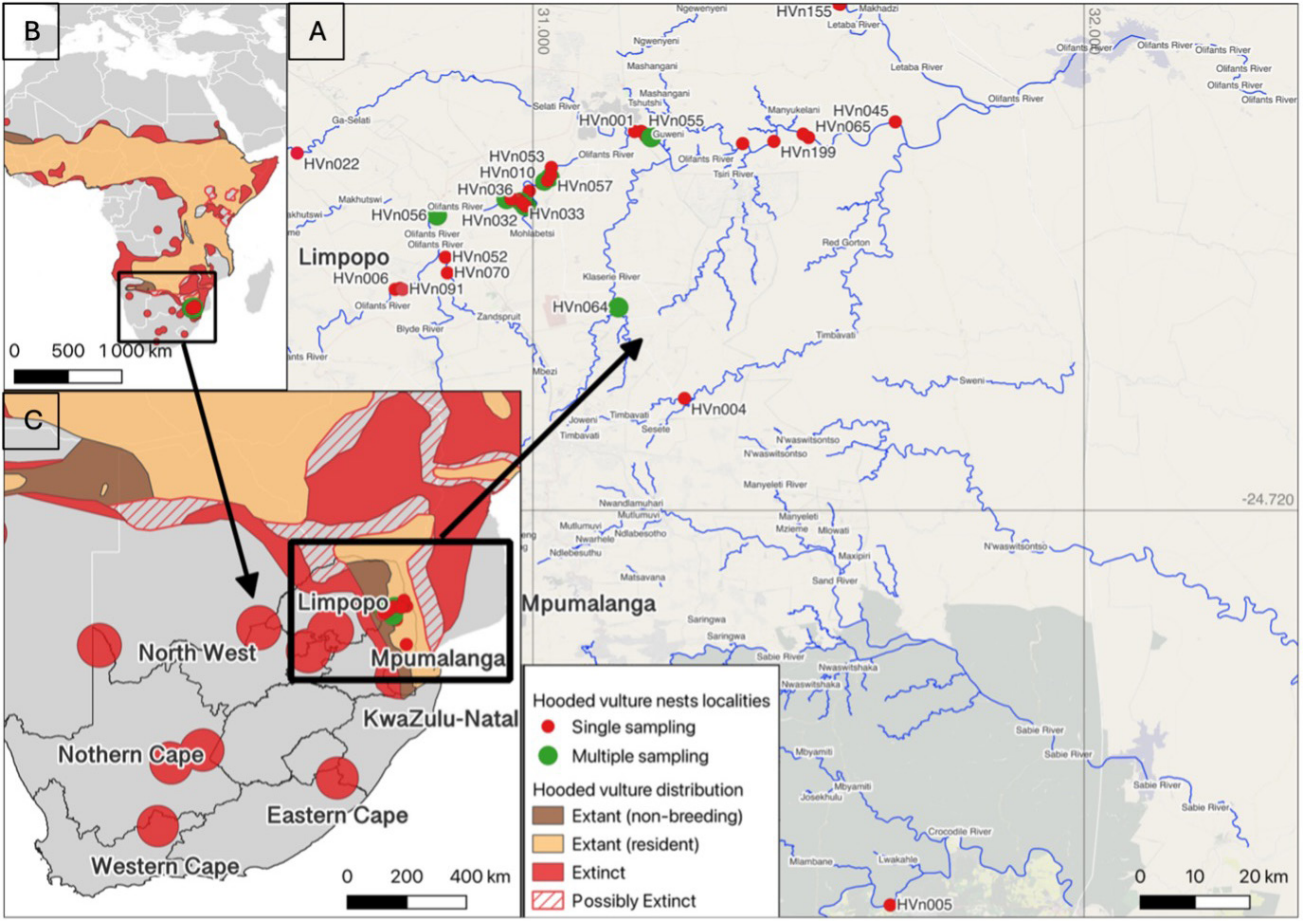
The localities of Hooded Vulture nests sampled in the present study (A). Nests were sampled between 2015 and 2019. The nests that were sampled once are shown by the red dots (label indicating the given nest label when sampled). The nests that were sampled over multiple years are indicated in green. The continental (B) and southern African distribution (C) of the species are also provided (BirdLife International 2022).

Sampling took place along a 94.06 km section loosely following the Olifants River, South Africa. The sampling area forms part of the core distribution of this species in South Africa, with the Olifants River being prime habitat for Hooded Vulture nesting. When possible, all nests that were found along the transect were sampled. Opportunistic sampling was done when nests were located in different areas in the Lowveld, with two nests sampled 67.84 km and 163.67 km away from the core distribution along the Olifants River. A total of 49 Hooded Vulture nests were sampled (Figure 1). Moulted feathers were collected underneath and inside nests during breeding seasons between 2015 and 2019. Nine nests were sampled over multiple years. This subset of the data (*n* = 42 genotypes) was used to assess nest turnover and determine if the same individuals were using the same nest over multiple years.

### 2.3 DNA Extraction and Microsatellite Amplification

The E.Z.N.A Tissue DNA kit (Omega Bio‐Tek, United States) was used for all DNA extractions. DNA was extracted from the calamus of feathers; modifications were made to the extraction protocol to ensure sufficient DNA yield from feathers. The modifications include incubating samples in proteinase K and lysis buffer for 24 h in a heated water bath (56°C). For the first 5 h, the samples were removed every hour to be vortexed. The lysate was incubated in B3 buffer for 45 min (70°C), and the final volume of pre‐warmed Buffer BE was added to the spin column, centrifuged, then reheated for 5 min and reapplied to the membrane before a final centrifuge step. All DNA extracts were stored at −20°C.

Fourteen microsatellite loci (Table 1) were used to calculate the relatedness of individual Hooded Vultures in this study. These microsatellite loci were used in previous studies of *Gyps coprotheres*, *Gypaetus barbatus* (Kleinhans and Willows‐Munro 2019; Streicher et al. 2021) and other vulture species (Çakmak et al. 2019; Davidović et al. 2020), which allows for the comparison of genetic diversity indices among different vulture species. The loci were amplified in five multiplex reactions using the Multiplex TEMPase PCR Kit (AMPLIQON, Denmark). Each forward primer was fluorescently labelled. The details of primers used in each multiplex, the fluorescent dye use and annealing temperatures are provided in Table 1.

**TABLE 1:** List of microsatellite loci used in genetic analyses of South African Hooded Vulture, primers used for amplification, fluorescent dyes used for tagging the forward primers, annealing temperature (Tm, °C) used in each reaction for the amplification of specific microsatellite loci with the combination of loci amplified together in multiplex reactions.

Locus name	Primer pair	Dye	Tm	Motif	Multiplex
BV2	F: CAGCATGTTATTTTGGCTGC	HEX	60	CA_11_	A
	R: TTGCTAAACCGGTTAGAAGTTG				
BV6	F: AATCTGCATCCCAGTTCTGC	HEX	60	CA_11_	D
	R: CCGGAGACTCTCAGAACTTAAC				
BV8	F: TGGCATGCTGCTATGAGAAC	FAM	60	CA_11_	C
	R: GTGCTTTGCATGCTTTTACTC				
BV9	F: ATCTAGGGACATCGAGGAGC	HEX	60	TA_6_ CA_11_	D
	R: ACAGGGATGCAGGTAAGCC				
BV11	F: TGTTTGCAAGCTGGAGACC	HEX	60	CA_22_	B
	R: AAAAGCCTTGGGGTAAGCAC				
BV12	F: TCAGGTTTTGACGACCTTCC	FAM	60	CA_15_	C
	R: GTGGTAACGGAGGAACAAGC				
BV13	F: AAAACAGAGTTTTCACATTTTCATAAG	FAM	60	CA_16_	A
	R: TTCAGGAAACAGAAGCATGAAC				
BV17	F: TGATGTGCAGATGCGTGAC	HEX	60	CA_11_	C
	R: GGACTCTGATGAAGCCAAGC				
BV20	F: GAACAGCACTGAACGTGAGC	HEX	58	CA_13_	E
	R: GTTTCTCCTGACAGTGAAATAACTC				
Gf3f3	F: GATCTTTCCCCTTCTGTG	TET	60	CT_10_	E
	R: TTCGTGCAGTGATGCTGGTG				
Gf3H3	F: GTAGAATAATTTGCTCCTGG	FAM	60	CT_12_	D
	R: GTGAAGGCACCTCATAGACA				
Gf8G	F: TGAGCAGGTGAGTCCAGAAG	FAM	60	CT_8_C TC_2_	B
	R: GCTCTCCTGTCATCTTGCAT				
Gf9C	F: GGTGGACATTACATACACTG	HEX	60	TC_10_ + CT_9_ C CA_5_T AC_4_	A
	R: CAAGGAATCTGGACTACTAA				
Gf11A4	F: GATCCCTTCCAACCGAAAAT	HEX	60	CTCTT_17_	C
	R: TGGTGACCAACGGAAGTGTG				

Each 10 μL reaction consisted of ~2–30 ng of DNA template, 5 μL of Multiplex TEMPase mix and 0.2 μL of 0.2 μM for each fluorescently tagged primer and purified water. The thermocycler parameters were as follows: initial denature at 94°C for 15 min, then followed by 94°C for denaturing for 30 s, 60°C for annealing for 1 min and then 72°C for elongation for 1 min for a total of 35 cycles. A final elongation at 72°C for 10 min completed the reactions with a hold at 4°C until samples were removed from the thermocycler. Negative controls were included in all sets of reactions. All amplified products were sent to the Central Analytic Facility at Stellenbosch University (South Africa) for fragment analyses. Genotype scoring was performed using GeneMarker v2.6.3 (Soft Genetics). To ensure genotype consistency, all samples were re‐amplified at least seven times and checked for consistency.

### 2.4 Data Analyses

Given that the primary source of DNA was from moulted feathers, an identification test was first done using Cervus v3.0.752 (Kalinowski, Taper, and Marshall 2007) to ensure duplicate samples collected from the same nest were not used in downstream analyses. The final dataset included 108 genotypes. Cervus was also used to estimate polymorphic information content (PIC) for each locus to ensure that loci were variable enough for the analyses among closely related individuals. Null allele frequencies were estimated using FreeNA (Chapuis and Estoup 2007). GenALEx v6.502 (Peakall and Smouse 2006) was used to assess the number of alleles (A) per loci and the number of effective alleles (*N*_e_). Universal Transverse Mercator grid data for each nest was obtained in QGIS v3.26 (QGIS Development Team 2021) then the geographical distance matrix between nests was calculated in GenALEx.

### 2.5 Relatedness of Individuals Across the Landscape

#### 2.5.1 Relatedness of Individuals Correlated to Geographic Distance Between Nesting Sites

The relatedness of Hooded Vulture individuals (*n* = 108) collected from the different nesting sites was estimated using two different methods in Coancestry v1.0.1.9 (Wang 2011). The internal individual relatedness coefficient was calculated for each individual using Lynch and Ritland (1999; LynchRD) and the triadic maximum likelihood (TrioML; Wang 2007) methods. The Lynch and Ritland test calculates relatedness based on the probability of alleles of a random locus being identical by descent (Lynch and Ritland 1999; Wang 2011). The triadic maximum likelihood method uses a maximum likelihood to calculate the relatedness between two individuals for a set of population allele frequencies (Wang 2007). These analyses were conducted without first‐order kin accounting for inbreeding and an error rate of 0.0005. Nei's genetic distance between individuals was also calculated in GenALEx.

Mantel tests (Mantel 1967) performed in GenALEx were conducted to determine if there was a significant correlation between relatedness (using LynchRD, TrioML and Nei's genetic distance) and geographical distance. To further assess the correlation between nest use and relatedness, spatial autocorrelation tests were also conducted in GenALEx, using 100 even distance classes of 2 km each (the closest distance between two nests) for 1000 permutations and 1000 bootstrap replicates of pairwise comparisons within each class. Bootstrap replicates provided a 95% confidence interval within each distance class.

To further assess fine‐scale population structure among individuals and their nest site location, the relatedness between the individuals breeding along the Olifants River (*n* = 99) and away from the Olifants River (*n* = 9) was compared. This was done by comparing the mean relatedness of individuals nesting along the river to individuals nesting away from the Olifants River, using a non‐parametric two‐sample Wilcoxon test in R v4.2.1 (2022‐06‐23) (R Core Team 2022).

To further visualise the genetic relationships between individuals, allele‐sharing networks were created using EDENetworks v2.18 (Kivelä, Arnaud‐Haond, and Saramäki 2015). In these networks, individuals are represented as nodes with connections between nodes weighted by their pairwise genetic distance (*F*_st_).

### 2.6 Nest Usage Over Time

Using the 42 genotypes from the nine nests that were sampled over multiple years, we used identification tests in Cervus to detect if the same genotypes were detected in feathers collected from the same nest over multiple years.

## 3 Results

Fourteen microsatellite markers were successfully amplified resulting in a final dataset including genotypes from 108 individuals. The PIC values (Table 2) indicated that eleven of the loci (BV2, BV13, Gf9C, BV11, BV12, Gf11A4, BV9, BV6, Gf3H3, BV20, Gf3f3) were highly informative (PIC > 0.5). Six of the loci (Gf8G, BV8, Gf11A4, BV9, BV20, Gf3f3) showed elevated levels of null alleles (*N*_0_ > 0.12). Overall, the Lowveld Hooded Vulture population showed a number of alleles per locus (A) ranging from 4 to 18 with a mean effective number of alleles (*N*_e_) of 3.442, with five loci having *N*_e_ > 4 (Gf9C, BV12, Gf11A4, BV6, Gf3H3).

**TABLE 2:** Summary showing the genetic diversity of microsatellite loci amplified from Hooded Vulture from South Africa sampled in the Lowveld area.

Locus	A	*N*_e_	*N*_0_	PIC
BV2	4	2.383	0.118	0.531
BV13	10	2.458	0.042	0.552
Gf9C	17	5.717	0.069	0.804
BV11	12	2.730	0.021	0.586
Gf8G	6	1.505	0.150	0.317
BV17	8	1.228	0.089	0.182
BV8	4	1.441	0.241	0.279
BV12	17	6.227	0.063	0.822
Gf11A4	9	4.902	0.292	0.767
BV9	9	2.944	0.155	0.621
BV6	18	6.340	0.065	0.825
Gf3H3	12	4.172	0.000	0.725
BV20	8	2.261	0.134	0.525
Gf3f3	6	3.881	0.145	0.699
Mean	10	3.442	0.113	—

*Note:* It shows the number of alleles (A), the number of effective alleles (*N*_e_), the null allele frequency (*N*_0_) and the polymorphic information content (PIC) of each locus.

### 3.1 Relatedness of Individuals Across the Landscape

**FIGURE 2:**
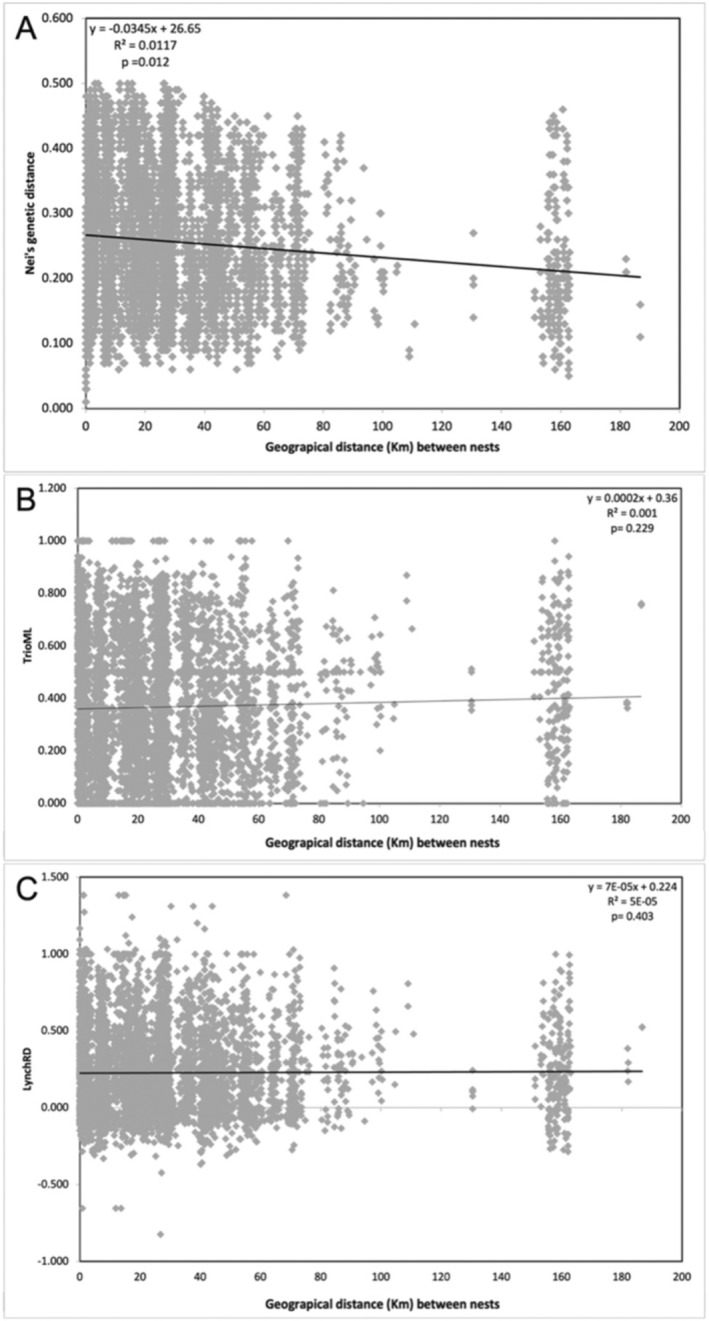
Mantel test plotting (A) Nei's genetic distance (*y*‐axis), (B) TrioML and (C) LynchRD relatedness coefficient against geographical distance (km) between Hooded Vulture nests (*x*‐axis) in the South African Lowveld region.

Nei's genetic distance between individuals ranged between 0.1 and 0.5 (Figure 2A). The Mantel test showed a significant negative correlation between geographical distance among nests and Nei's genetic distance among individuals (*r* = −0.108, *R* ^2^ = 0.0117, *p* = 0.012) (Figure 4A). However, when Mantel tests were performed using the TrioML or LynchRD relatedness coefficient values (Figure 2B,C) no significant correlations were found (TrioML: *r* = 0.031, *R* ^2^ = 0.001, *p*‐value = 0.229; LynchRD: *r* = 0.007, *R* ^2^ = 0.0005, *p*‐value = 0.409).

**FIGURE 3:**
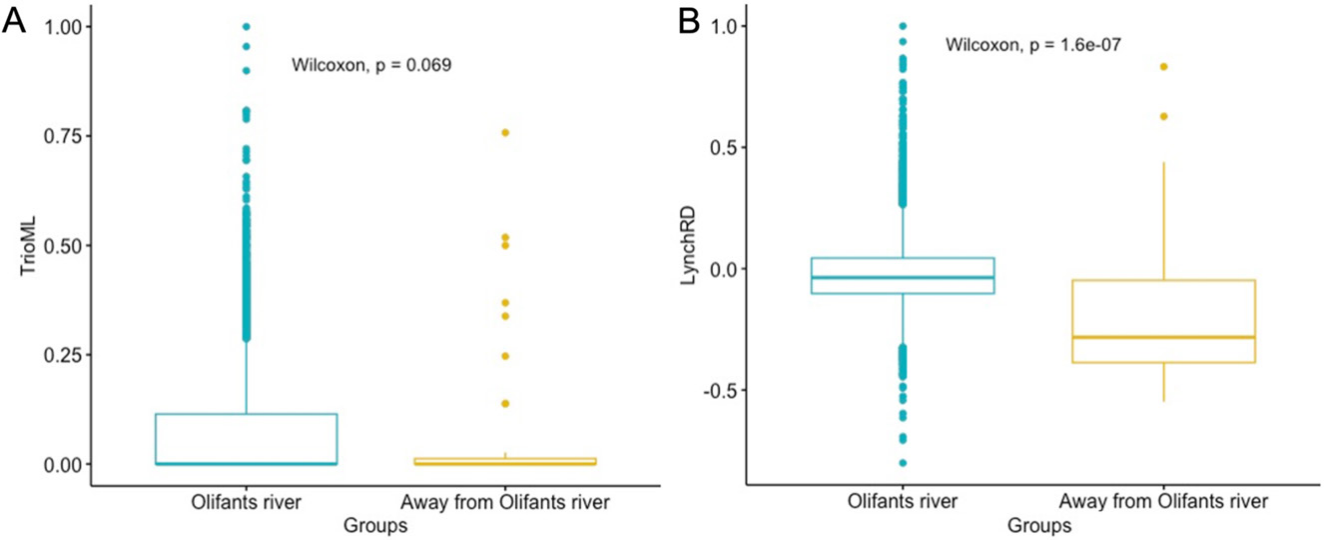
The spatial autocorrelation analysis of individuals from the South African Lowveld Hooded Vulture population. The solid blue line indicates the autocorrelation coefficient data with a 95% confidence interval indicated by the black error bars, and the dotted red line indicates the 95% confidence interval around the null hypothesis.

Interestingly, spatial autocorrelation analysis indicated a significant correlation between genetic distance among individuals and geographical distance among nests for some distance classes. These classes included distance class 2–6 km, 10–18 km, 38–42 km, 50–58 km, 98–102 km, 110–114 km, 150–158 km and 185–190 km (Figure 3). This suggests that individuals in these classes were genetically more similar than expected by chance. In contrast, some distance classes indicated a negative correlation (30–34 km, 46–50 km, 74–78 km, 78–86 km and 90–94 km) (Figure 3).

**FIGURE 4:**
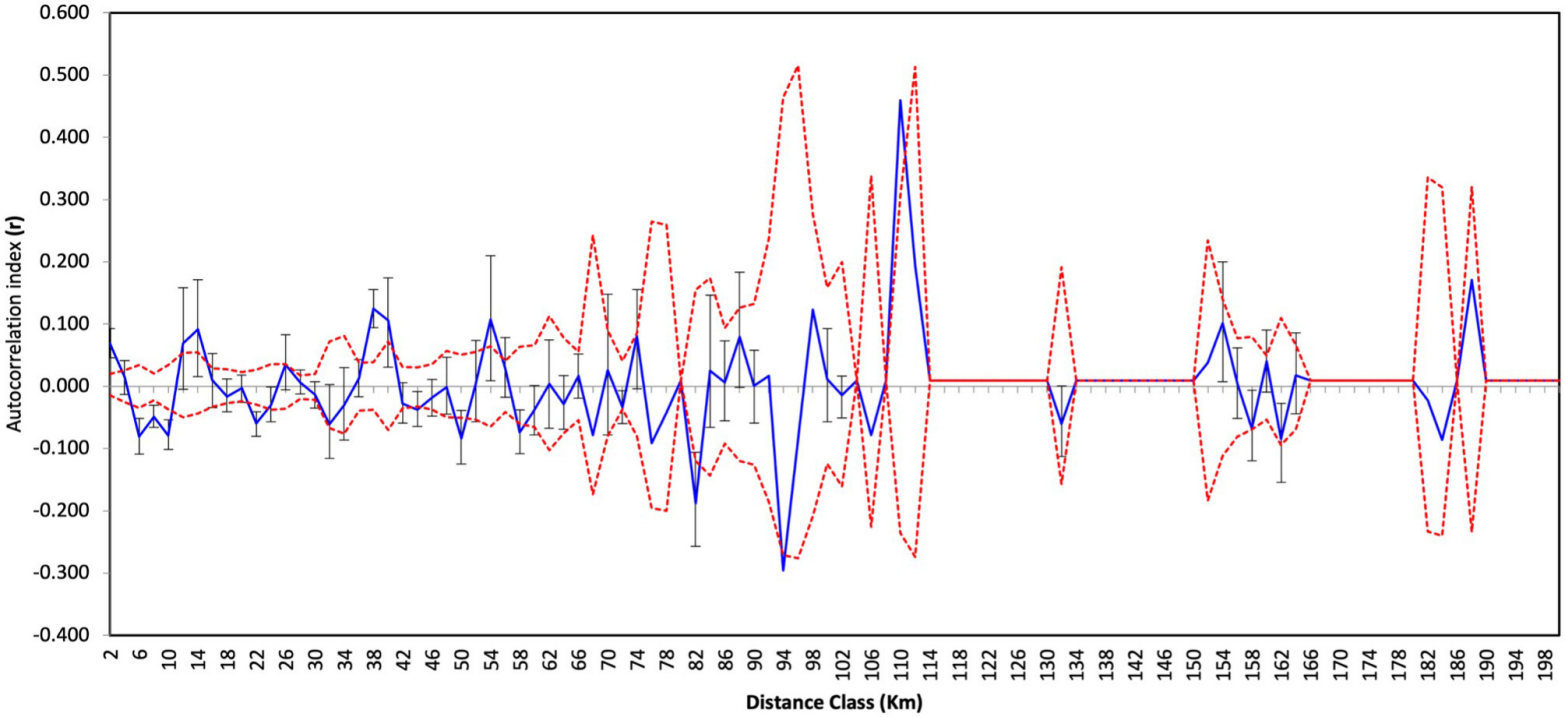
Boxplots showing the relatedness between Hooded Vulture individuals breeding along the Olifants River and away from the Olifants River for relatedness coefficient estimator TrioML (A) and LynchRD (B). The text insert indicates the *p*‐value of the two‐sample Wilcoxon test, for a difference in means.

The Wilcoxon test did not indicate a significant difference between the mean relatedness for TrioML relatedness measures for individuals breeding along the Olifants River compared to individuals breeding away from the Olifants River (*p*‐value = 0.069) (Figure 4A). The Wilcoxon test did, however, indicate a significant difference in the mean relatedness between individuals breeding away from the Olifants River and individuals breeding along the Olifants River (*p*‐value = 0.00000016) for the LynchRD relatedness coefficient suggesting that individuals tend to be more related around the Olifants River (Figure 4B).

**FIGURE 5:**
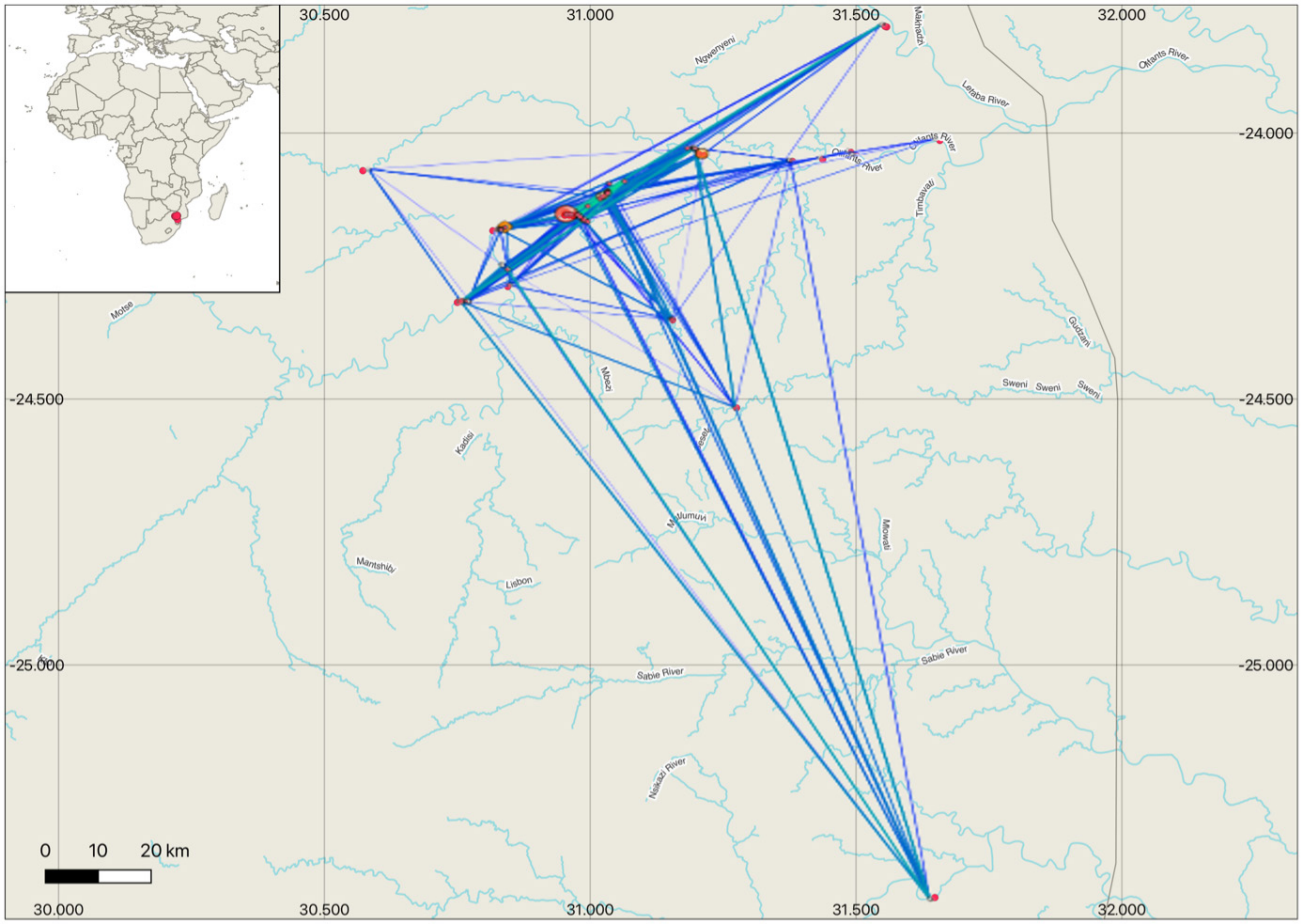
Locations of some Hooded Vulture nests in the north‐eastern part of southern Africa. The relatedness network (constructed using EDENetworks) has been overlayed.

As expected, the relatedness network constructed for the Lowveld Hooded Vulture population was well connected, with many individuals sharing alleles (Figure 5). Interestingly, individuals breeding along the Olifants River share more alleles, while individuals nesting further away from the river share less genetic similarity.

### 3.2 Nest Usage Over Time

Despite surveying nine nests over 5 years, we found no evidence that the same individual used the same nest across different breeding seasons. No identical genotypes were recovered from the same nest across different years.

## 4 Discussion

Theory would suggest that obligate scavengers such as Hooded Vultures that are social feeders and rely on patchy and unpredictable food sources would gain benefit from communal living. By taking advantage of the searching power of many, individuals who were unsuccessful at foraging could benefit from following more successful individuals to food. If Hooded Vulture colonies are operating as ‘food finding information hubs’, then we expect higher levels of relatedness between individuals within a colony, as relatedness increases the tolerance between individuals to allow food‐finding information to be shared (Ward and Zahavi 1973; Erwin 1978; Waltz 1982; Dermody, Tanner, and Jackson 2011). This increases the inclusive fitness of individuals within the colony (Hamilton 1964). However, the negative correlation shown by the Mantel test in this study indicates that nesting sites around the Olifants River may not be communal breeding sites that serve as a food‐finding communication sharing hubs. This type of behaviour where breeding colonies are formed is seen in White‐backed Vultures (*Gyps africanus*), where nests occur in colonies rather than single nesting sites (Johnson and Murn 2023). These colony nesting sites in White‐backed Vultures showed positive effects on the breeding success of these birds (Johnson and Murn 2023). Similar observations have been made in Griffon Vultures (*Gyps fulvus*), where informed individuals are followed to food resources by uninformed individuals (Harel et al. 2017).

Natal distribution has been shown to influence nest selection in the White‐tailed eagle (*Haliaeetus albicilla*), with indivduals born in high‐density areas showing smaller natal distribution; however, once mature these birds tend to settle in low‐density areas (Çakmak et al. 2019). Natal distribution has also been shown to influence population structuring in the Cinereous vulture (*Aegypius monachus*) in Turkey, as levels of relatedness within the colonies were not high due to birds frequently moving away from natal colonies to breed. Thus, with the limited knowledge of the social behaviour of Hooded Vultures natal distribution could be directly influenced by relatedness, which would lead to the negative correlation between relatedness and distance between nests seen in this study.

### 4.1 Nest Usage Over Time

The breeding success of the Hooded Vulture is of conservation importance given the small size of the population in Southern Africa. Despite previous studies noting that Hooded Vultures in southern Africa are resident and generally sedentary (Ferguson‐Lees and Christie 2001; Reading et al. 2019; Thompson et al. 2020), no individuals in the present study reused nests over a sampling period of 5 years. A similar trend has also been observed in other raptor species such as booted eagle (*Aquila pennata*) and common buzzard (*Buteo buteo*; Jiménez‐Franco, Martínez, and Calvo 2014). This suggests that although the species is fairly sedentary within the area, pairs may frequently move to find better breeding habitats. This could be based on the success of a specific nesting area or how often the nest is visited by other species (such as Egyptian geese, *Alopochen aegyptiaca*) that interfere with breeding. A similar study conducted on White‐tailed eagles showed that these birds of prey often use alternative nests for breeding (Bulut et al. 2016).

The high density of Hooded Vulture nests found along the Olifants River could be driven by habitat preference. Competition for nests could be a key consideration in the management of this vulnerable vulture population. Overall this study shows that the Olifants River is an important breeding site for the Lowveld Hooded Vulture population and highlights the need for more studies into this species' life history and breeding behaviour. Furthermore, this study highlights the need for increased conservation of critical breeding habitat around the Olifants River. Increasing habitat restoration and tree planting efforts to increase nesting trees available for this species may be a valuable strategy to improve population numbers. Conservation actions that could be implemented to conserve the species could include captive breeding programs, which would act as a supplementary source of genetic diversity. Important information on Habitat preference should be used to guide the establishment of Hooded Vulture breeding populations outside of the Kruger National Park.

## Author Contributions

**Rynhardt Le Roux:** conceptualization (equal), data curation (equal), formal analysis (equal), investigation (equal), writing – original draft (equal). **Lindy J. Thompson:** conceptualization (equal), data curation (equal), formal analysis (equal), project administration (equal), resources (equal), supervision (equal), writing – review and editing (equal). **Bettine Jansen van Vuuren:** formal analysis (equal), supervision (equal), writing – review and editing (equal). **Sandi Willows‐Munro:** conceptualization (equal), data curation (equal), formal analysis (equal), funding acquisition (equal), project administration (equal), resources (equal), supervision (equal), writing – review and editing (equal).

## Conflicts of Interest

The authors declare no conflicts of interest.

## Data Availability

The data analysed in this study will be made available through FigShare (https://url.za.m.mimecastprotect.com/s/ZoQsCWnBo2frWKwgH664sl?domain=figshare.com).
